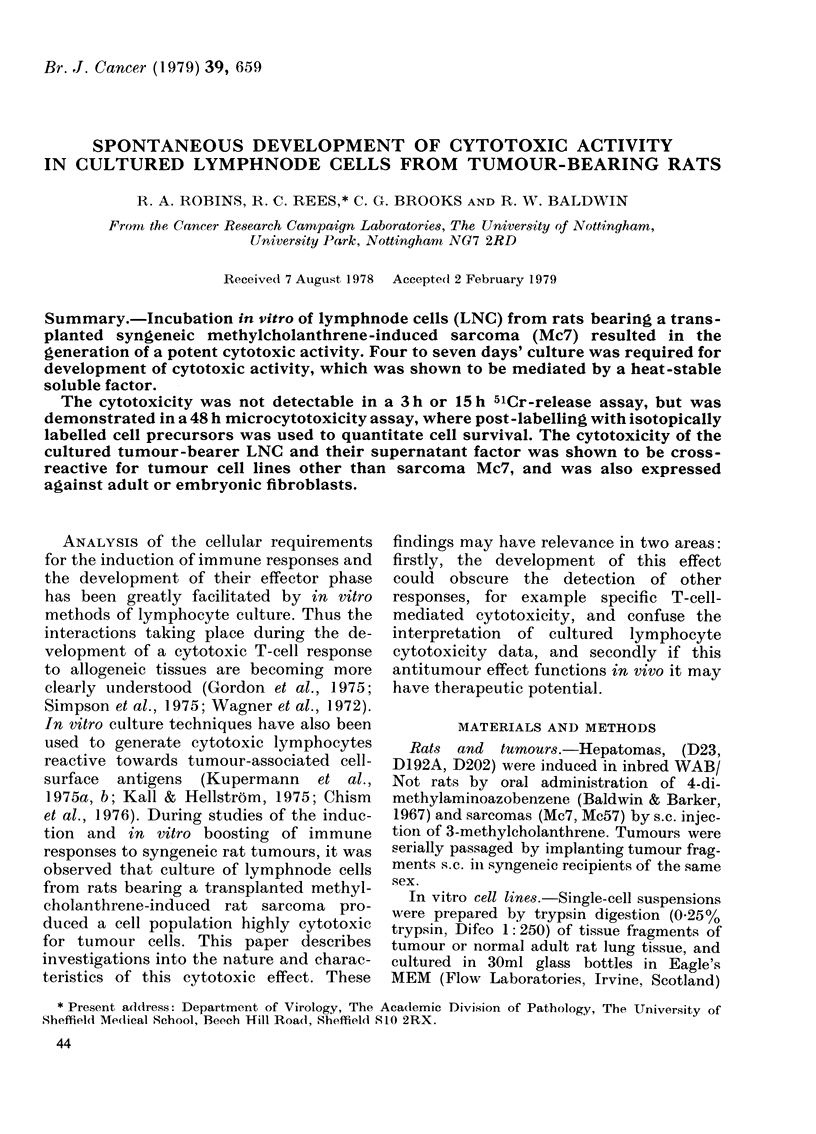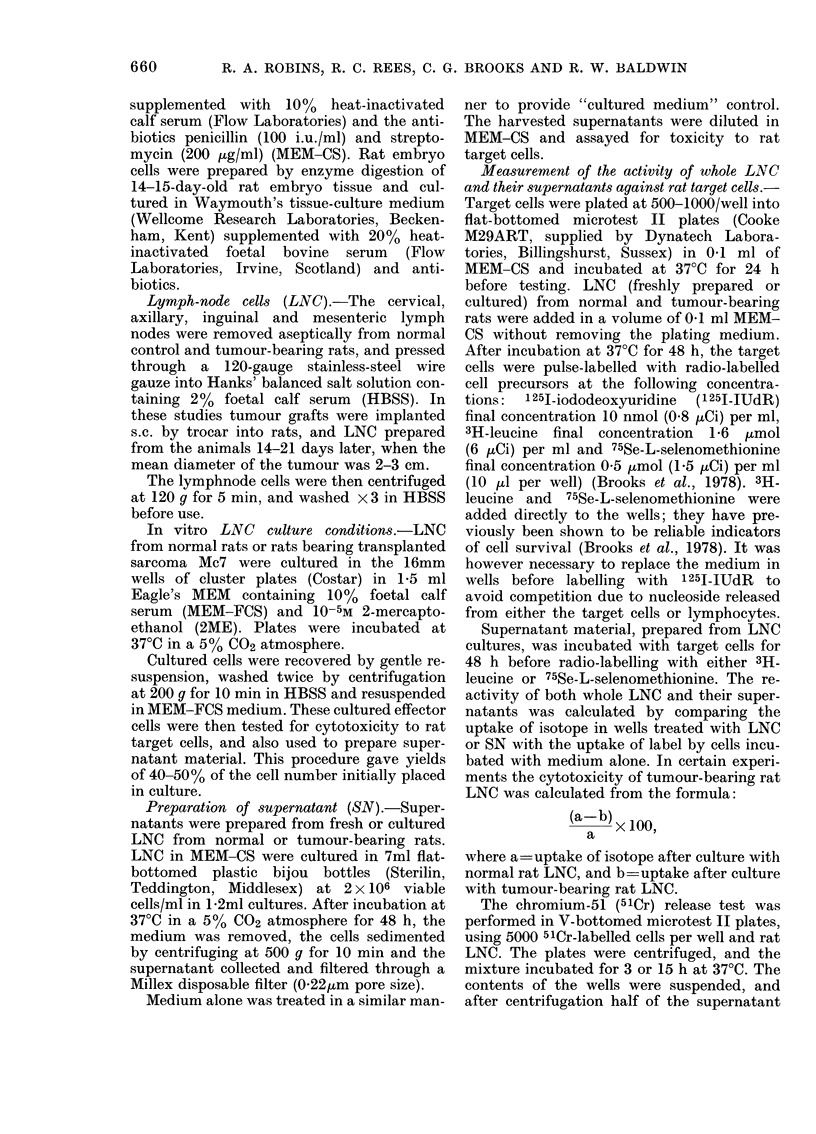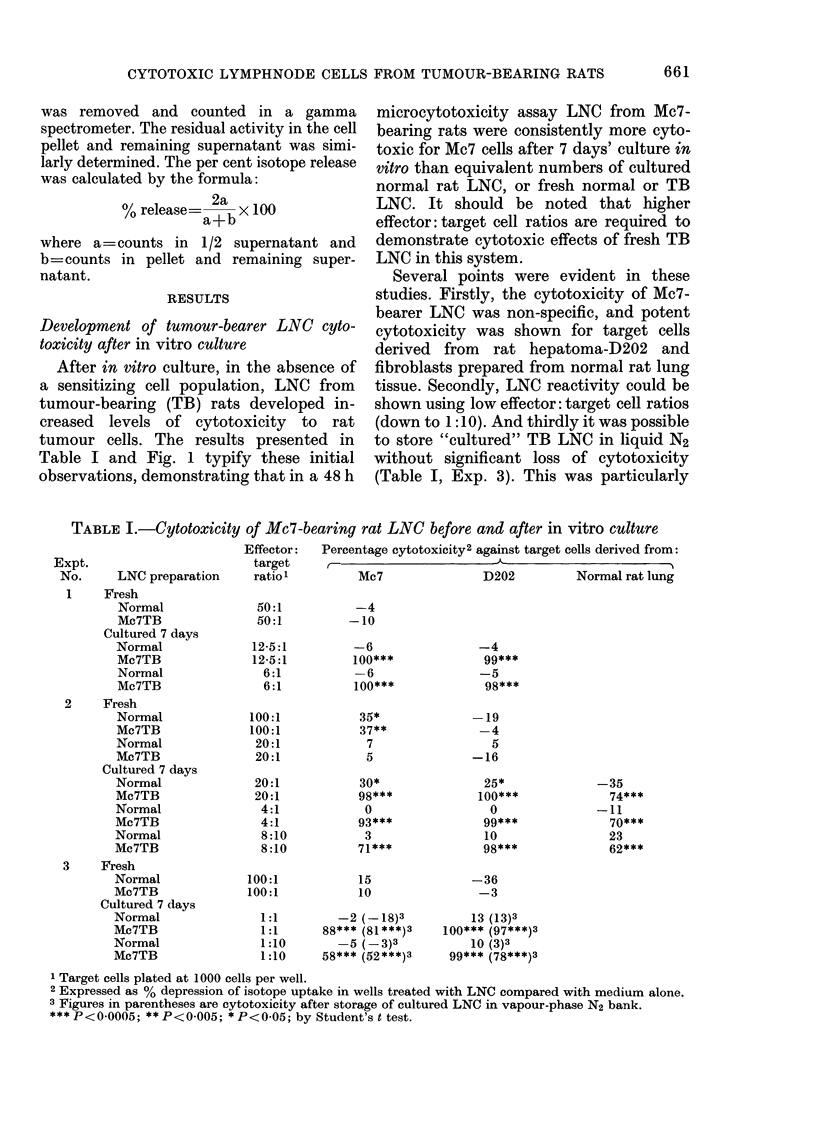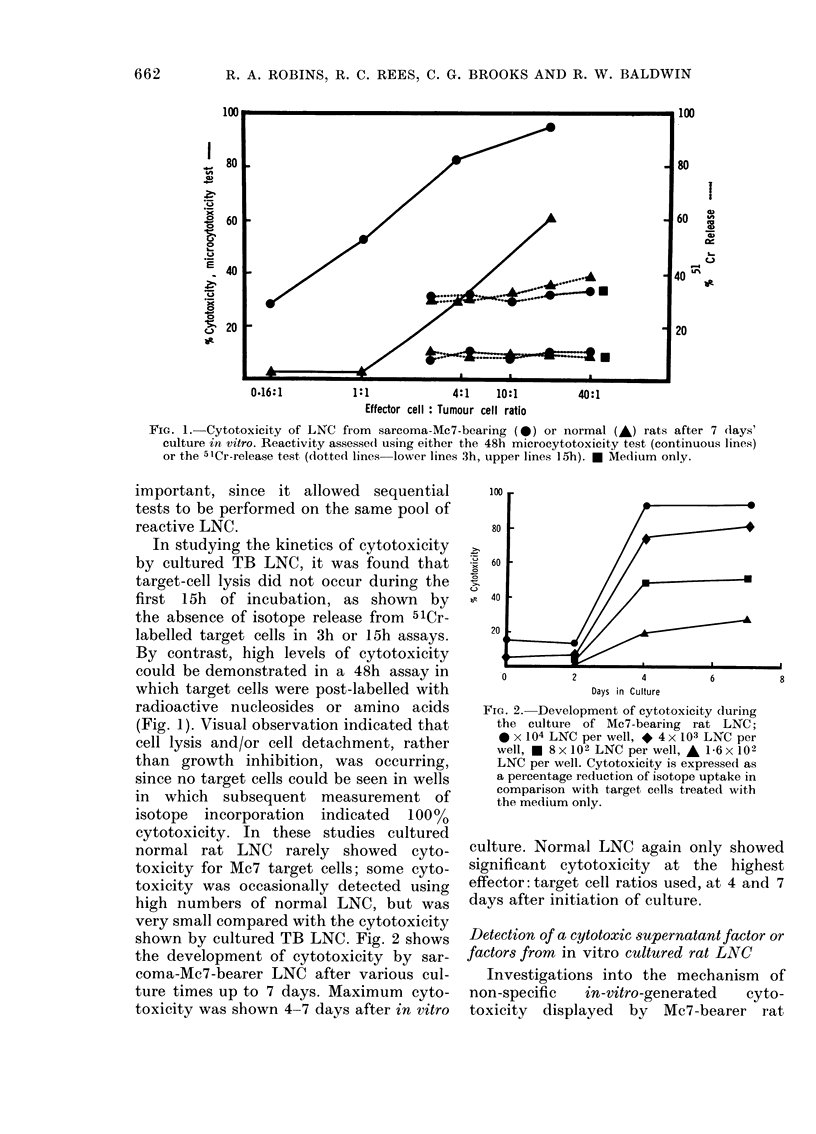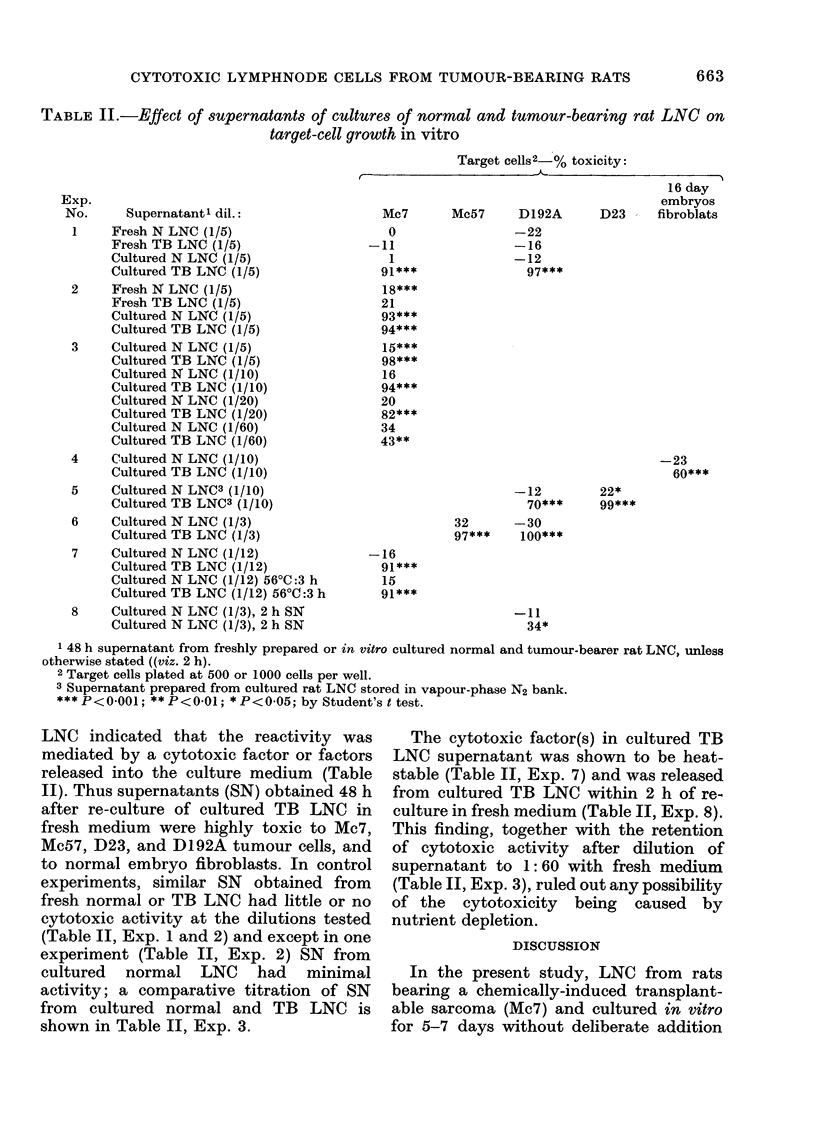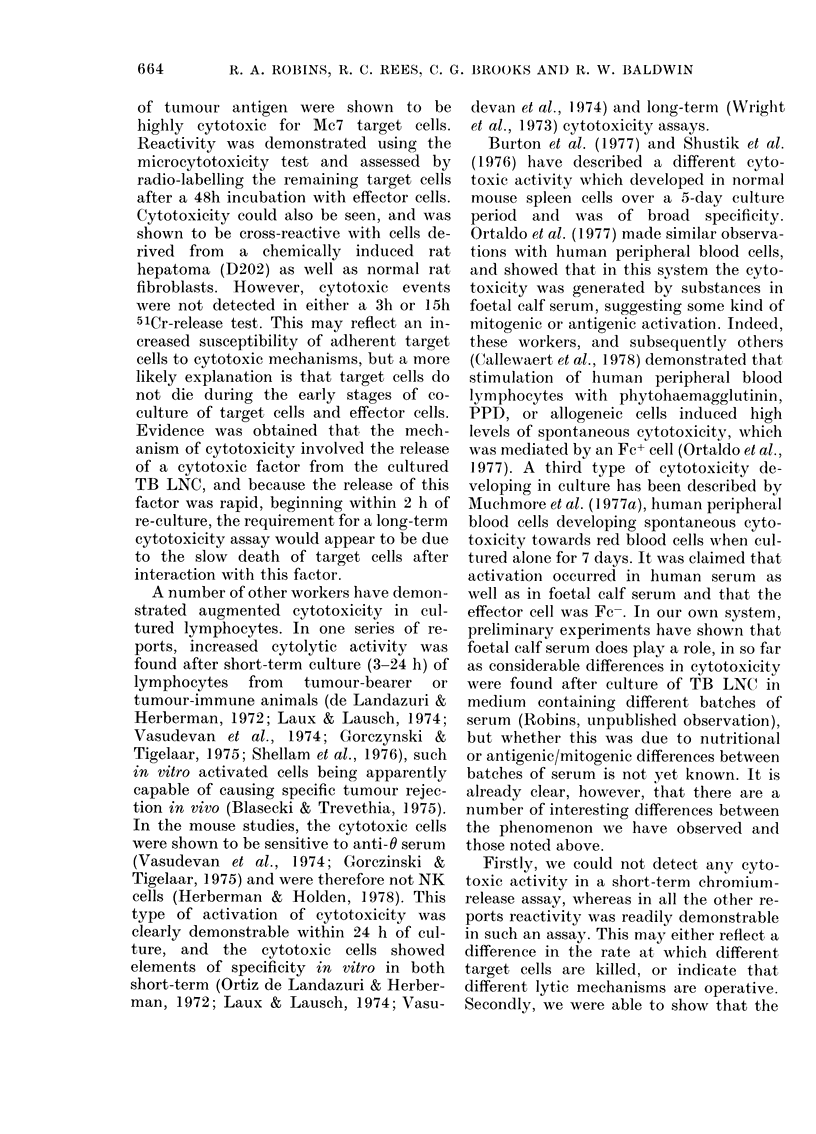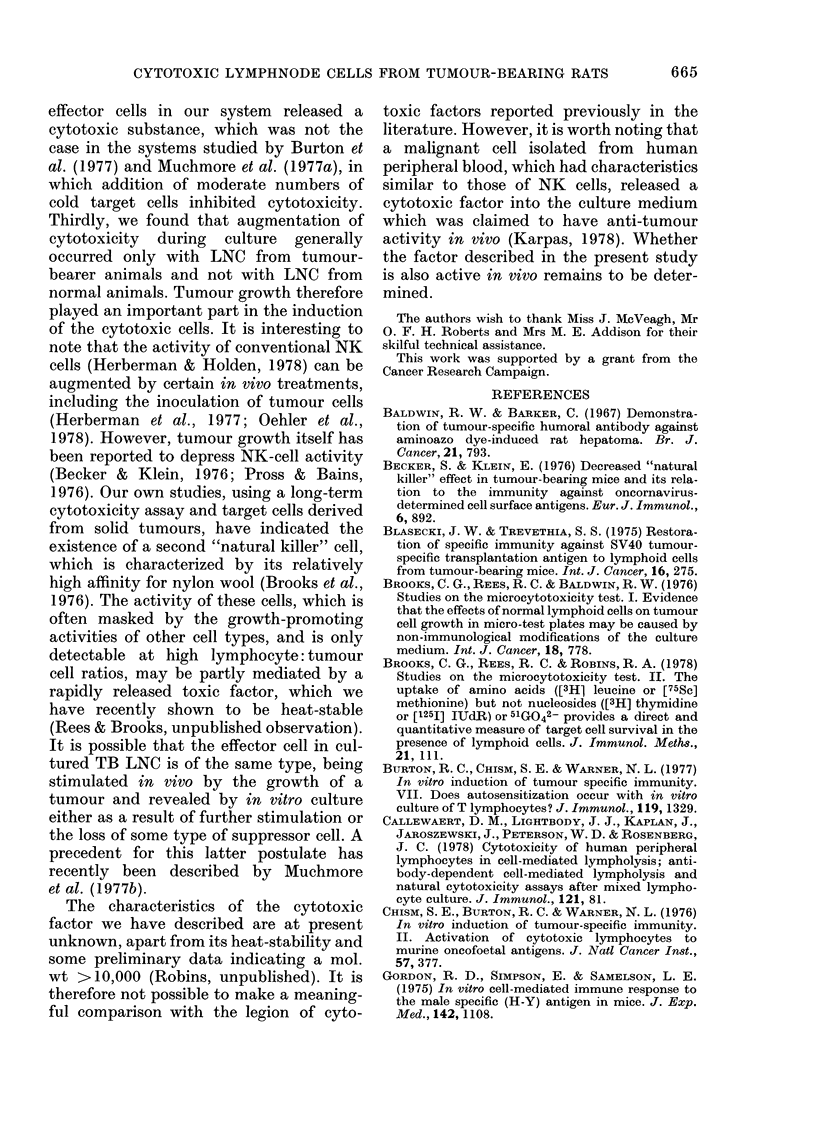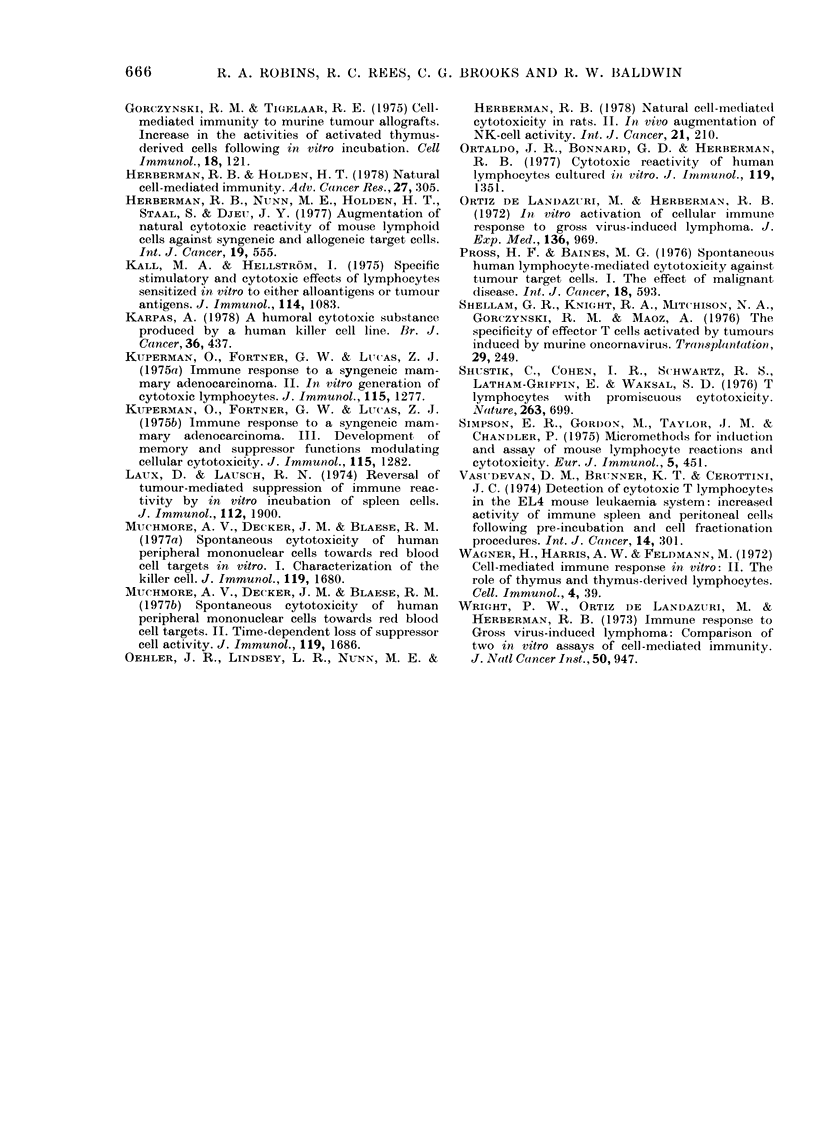# Spontaneous development of cytotoxic activity in cultured lymphnode cells from tumour-bearing rats.

**DOI:** 10.1038/bjc.1979.117

**Published:** 1979-06

**Authors:** R. A. Robins, R. C. Rees, C. G. Brooks, R. W. Baldwin

## Abstract

Incubation in vitro of lymphnode cells (LNC) from rats bearing a transplanted syngeneic methylcholanthrene-induced sarcoma (Mc7) resulted in the generation of a potent cytotoxic activity. Four to seven days' culture was required for development of cytotoxic activity, which was shown to be mediated by a heat-stable soluble factor. The cytotoxicity was not detectable in a 3 h or 15 h 51Cr-release assay, but was demonstrated in a 48 h microcytotoxicity assay, where post-labeling with isotopically labelled cell precursors was used to quantitate cell survival. The cytotoxicity of the cultured tumour-bearer LNC and their supernatant factor was shown to be cross-reactive for tumour cell lines other than sarcoma Mc7, and was also expressed against adult or embryonic fibroblasts.


*Br. J. Cancer* (1979) **39**, 659

### SPONTANEOUS DEVELOPMENT OF CYTOTOXIC ACTIVITY IN CULTURED LYMPHNODE CELLS FROM TUMOUR-BEARING RATS

R. A. ROBINS, R. C. REES,* C. G. BROOKS AND R. W. BALDWIN

*From the Cancer Research Campaign Laboratories, The University of Nottingham, University Park, Nottingham NG7 2RD*

Received 7 August 1978 Accepted 2 February 1979

**Summary.—Incubation *in vitro* of lymphnode cells (LNC) from rats bearing a transplanted syngeneic methylcholanthrene-induced sarcoma (Mc7) resulted in the generation of a potent cytotoxic activity. Four to seven days' culture was required for development of cytotoxic activity, which was shown to be mediated by a heat-stable soluble factor.**

**The cytotoxicity was not detectable in a 3 h or 15 h $^{51}$Cr-release assay, but was demonstrated in a 48 h microcytotoxicity assay, where post-labelling with isotopically labelled cell precursors was used to quantitate cell survival. The cytotoxicity of the cultured tumour-bearer LNC and their supernatant factor was shown to be cross-reactive for tumour cell lines other than sarcoma Mc7, and was also expressed against adult or embryonic fibroblasts.**

ANALYSIS of the cellular requirements for the induction of immune responses and the development of their effector phase has been greatly facilitated by *in vitro* methods of lymphocyte culture. Thus the interactions taking place during the development of a cytotoxic T-cell response to allogeneic tissues are becoming more clearly understood (Gordon *et al.*, 1975; Simpson *et al.*, 1975; Wagner *et al.*, 1972). *In vitro* culture techniques have also been used to generate cytotoxic lymphocytes reactive towards tumour-associated cell-surface antigens (Kupermann *et al.*, 1975*a*, *b*; Kall & Hellström, 1975; Chism *et al.*, 1976). During studies of the induction and *in vitro* boosting of immune responses to syngeneic rat tumours, it was observed that culture of lymphnode cells from rats bearing a transplanted methylcholanthrene-induced rat sarcoma produced a cell population highly cytotoxic for tumour cells. This paper describes investigations into the nature and characteristics of this cytotoxic effect. These findings may have relevance in two areas: firstly, the development of this effect could obscure the detection of other responses, for example specific T-cell-mediated cytotoxicity, and confuse the interpretation of cultured lymphocyte cytotoxicity data, and secondly if this antitumour effect functions *in vivo* it may have therapeutic potential.

### MATERIALS AND METHODS

*Rats and tumours.*—Hepatomas, (D23, D192A, D202) were induced in inbred WAB/Not rats by oral administration of 4-dimethylaminoazobenzene (Baldwin & Barker, 1967) and sarcomas (Mc7, Mc57) by s.c. injection of 3-methylcholanthrene. Tumours were serially passaged by implanting tumour fragments s.c. in syngeneic recipients of the same sex.

In vitro *cell lines.*—Single-cell suspensions were prepared by trypsin digestion (0·25% trypsin, Difco 1:250) of tissue fragments of tumour or normal adult rat lung tissue, and cultured in 30ml glass bottles in Eagle's MEM (Flow Laboratories, Irvine, Scotland)

* Present address: Department of Virology, The Academic Division of Pathology, The University of Sheffield Medical School, Beech Hill Road, Sheffield S10 2RX.

supplemented with 10% heat-inactivated calf serum (Flow Laboratories) and the antibiotics penicillin (100 i.u./ml) and streptomycin (200 μg/ml) (MEM–CS). Rat embryo cells were prepared by enzyme digestion of 14–15-day-old rat embryo tissue and cultured in Waymouth's tissue-culture medium (Wellcome Research Laboratories, Beckenham, Kent) supplemented with 20% heat-inactivated foetal bovine serum (Flow Laboratories, Irvine, Scotland) and antibiotics.

*Lymph-node cells* (*LNC*).—The cervical, axillary, inguinal and mesenteric lymph nodes were removed aseptically from normal control and tumour-bearing rats, and pressed through a 120-gauge stainless-steel wire gauze into Hanks' balanced salt solution containing 2% foetal calf serum (HBSS). In these studies tumour grafts were implanted s.c. by trocar into rats, and LNC prepared from the animals 14–21 days later, when the mean diameter of the tumour was 2–3 cm.

The lymphnode cells were then centrifuged at 120 *g* for 5 min, and washed ×3 in HBSS before use.

In vitro *LNC culture conditions*.—LNC from normal rats or rats bearing transplanted sarcoma Mc7 were cultured in the 16mm wells of cluster plates (Costar) in 1·5 ml Eagle's MEM containing 10% foetal calf serum (MEM–FCS) and $10^{-5}$M 2-mercaptoethanol (2ME). Plates were incubated at 37°C in a 5% $CO_2$ atmosphere.

Cultured cells were recovered by gentle resuspension, washed twice by centrifugation at 200 *g* for 10 min in HBSS and resuspended in MEM–FCS medium. These cultured effector cells were then tested for cytotoxicity to rat target cells, and also used to prepare supernatant material. This procedure gave yields of 40–50% of the cell number initially placed in culture.

*Preparation of supernatant* (*SN*).—Supernatants were prepared from fresh or cultured LNC from normal or tumour-bearing rats. LNC in MEM–CS were cultured in 7ml flat-bottomed plastic bijou bottles (Sterilin, Teddington, Middlesex) at $2\times10^6$ viable cells/ml in 1·2ml cultures. After incubation at 37°C in a 5% $CO_2$ atmosphere for 48 h, the medium was removed, the cells sedimented by centrifuging at 500 *g* for 10 min and the supernatant collected and filtered through a Millex disposable filter (0·22μm pore size).

Medium alone was treated in a similar manner to provide "cultured medium" control. The harvested supernatants were diluted in MEM–CS and assayed for toxicity to rat target cells.

*Measurement of the activity of whole LNC and their supernatants against rat target cells.*—Target cells were plated at 500–1000/well into flat-bottomed microtest II plates (Cooke M29ART, supplied by Dynatech Laboratories, Billingshurst, Sussex) in 0·1 ml of MEM–CS and incubated at 37°C for 24 h before testing. LNC (freshly prepared or cultured) from normal and tumour-bearing rats were added in a volume of 0·1 ml MEM–CS without removing the plating medium. After incubation at 37°C for 48 h, the target cells were pulse-labelled with radio-labelled cell precursors at the following concentrations: $^{125}$I-iododeoxyuridine ($^{125}$I-IUdR) final concentration 10 nmol (0·8 μCi) per ml, $^{3}$H-leucine final concentration 1·6 μmol (6 μCi) per ml and $^{75}$Se-L-selenomethionine final concentration 0·5 μmol (1·5 μCi) per ml (10 μl per well) (Brooks *et al.*, 1978). $^{3}$H-leucine and $^{75}$Se-L-selenomethionine were added directly to the wells; they have previously been shown to be reliable indicators of cell survival (Brooks *et al.*, 1978). It was however necessary to replace the medium in wells before labelling with $^{125}$I-IUdR to avoid competition due to nucleoside released from either the target cells or lymphocytes.

Supernatant material, prepared from LNC cultures, was incubated with target cells for 48 h before radio-labelling with either $^{3}$H-leucine or $^{75}$Se-L-selenomethionine. The reactivity of both whole LNC and their supernatants was calculated by comparing the uptake of isotope in wells treated with LNC or SN with the uptake of label by cells incubated with medium alone. In certain experiments the cytotoxicity of tumour-bearing rat LNC was calculated from the formula:

$$\frac{(a-b)}{a}\times 100,$$

where a=uptake of isotope after culture with normal rat LNC, and b=uptake after culture with tumour-bearing rat LNC.

The chromium-51 ($^{51}$Cr) release test was performed in V-bottomed microtest II plates, using 5000 $^{51}$Cr-labelled cells per well and rat LNC. The plates were centrifuged, and the mixture incubated for 3 or 15 h at 37°C. The contents of the wells were suspended, and after centrifugation half of the supernatant was removed and counted in a gamma spectrometer. The residual activity in the cell pellet and remaining supernatant was similarly determined. The per cent isotope release was calculated by the formula:

$$\% \text{ release} = \frac{2a}{a+b} \times 100$$

where a = counts in 1/2 supernatant and b = counts in pellet and remaining supernatant.

### RESULTS

### *Development of tumour-bearer LNC cytotoxicity after* in vitro *culture*

After *in vitro* culture, in the absence of a sensitizing cell population, LNC from tumour-bearing (TB) rats developed increased levels of cytotoxicity to rat tumour cells. The results presented in Table I and Fig. 1 typify these initial observations, demonstrating that in a 48 h microcytotoxicity assay LNC from Mc7-bearing rats were consistently more cytotoxic for Mc7 cells after 7 days' culture *in vitro* than equivalent numbers of cultured normal rat LNC, or fresh normal or TB LNC. It should be noted that higher effector: target cell ratios are required to demonstrate cytotoxic effects of fresh TB LNC in this system.

Several points were evident in these studies. Firstly, the cytotoxicity of Mc7-bearer LNC was non-specific, and potent cytotoxicity was shown for target cells derived from rat hepatoma-D202 and fibroblasts prepared from normal rat lung tissue. Secondly, LNC reactivity could be shown using low effector: target cell ratios (down to 1:10). And thirdly it was possible to store "cultured" TB LNC in liquid $N_2$ without significant loss of cytotoxicity (Table I, Exp. 3). This was particularly

TABLE I.—*Cytotoxicity of Mc7-bearing rat LNC before and after* in vitro *culture*

| Expt. No. | LNC preparation | Effector: target ratio[1] | Percentage cytotoxicity[2] against target cells derived from: Mc7 | D202 | Normal rat lung |
|---|---|---|---|---|---|
| 1 | Fresh | | | | |
| | Normal | 50:1 | −4 | | |
| | Mc7TB | 50:1 | −10 | | |
| | Cultured 7 days | | | | |
| | Normal | 12·5:1 | −6 | −4 | |
| | Mc7TB | 12·5:1 | 100*** | 99*** | |
| | Normal | 6:1 | −6 | −5 | |
| | Mc7TB | 6:1 | 100*** | 98*** | |
| 2 | Fresh | | | | |
| | Normal | 100:1 | 35* | −19 | |
| | Mc7TB | 100:1 | 37** | −4 | |
| | Normal | 20:1 | 7 | 5 | |
| | Mc7TB | 20:1 | 5 | −16 | |
| | Cultured 7 days | | | | |
| | Normal | 20:1 | 30* | 25* | −35 |
| | Mc7TB | 20:1 | 98*** | 100*** | 74*** |
| | Normal | 4:1 | 0 | 0 | −11 |
| | Mc7TB | 4:1 | 93*** | 99*** | 70*** |
| | Normal | 8:10 | 3 | 10 | 23 |
| | Mc7TB | 8:10 | 71*** | 98*** | 62*** |
| 3 | Fresh | | | | |
| | Normal | 100:1 | 15 | −36 | |
| | Mc7TB | 100:1 | 10 | −3 | |
| | Cultured 7 days | | | | |
| | Normal | 1:1 | −2 (−18)[3] | 13 (13)[3] | |
| | Mc7TB | 1:1 | 88*** (81***)[3] | 100*** (97***)[3] | |
| | Normal | 1:10 | −5 (−3)[3] | 10 (3)[3] | |
| | Mc7TB | 1:10 | 58*** (52***)[3] | 99*** (78***)[3] | |

[1] Target cells plated at 1000 cells per well.
[2] Expressed as % depression of isotope uptake in wells treated with LNC compared with medium alone.
[3] Figures in parentheses are cytotoxicity after storage of cultured LNC in vapour-phase $N_2$ bank.
*** $P<0.0005$; ** $P<0.005$; * $P<0.05$; by Student's $t$ test.

FIG. 1.—Cytotoxicity of LNC from sarcoma-Mc7-bearing (●) or normal (▲) rats after 7 days' culture *in vitro*. Reactivity assessed using either the 48h microcytotoxicity test (continuous lines) or the $^{51}$Cr-release test (dotted lines—lower lines 3h, upper lines 15h). ■ Medium only.

important, since it allowed sequential tests to be performed on the same pool of reactive LNC.

In studying the kinetics of cytotoxicity by cultured TB LNC, it was found that target-cell lysis did not occur during the first 15h of incubation, as shown by the absence of isotope release from $^{51}$Cr-labelled target cells in 3h or 15h assays. By contrast, high levels of cytotoxicity could be demonstrated in a 48h assay in which target cells were post-labelled with radioactive nucleosides or amino acids (Fig. 1). Visual observation indicated that cell lysis and/or cell detachment, rather than growth inhibition, was occurring, since no target cells could be seen in wells in which subsequent measurement of isotope incorporation indicated 100% cytotoxicity. In these studies cultured normal rat LNC rarely showed cytotoxicity for Mc7 target cells; some cytotoxicity was occasionally detected using high numbers of normal LNC, but was very small compared with the cytotoxicity shown by cultured TB LNC. Fig. 2 shows the development of cytotoxicity by sarcoma-Mc7-bearer LNC after various culture times up to 7 days. Maximum cytotoxicity was shown 4–7 days after *in vitro*

FIG. 2.—Development of cytotoxicity during the culture of Mc7-bearing rat LNC; ● × $10^4$ LNC per well, ◆ $4 \times 10^3$ LNC per well, ■ $8 \times 10^2$ LNC per well, ▲ $1{\cdot}6 \times 10^2$ LNC per well. Cytotoxicity is expressed as a percentage reduction of isotope uptake in comparison with target cells treated with the medium only.

culture. Normal LNC again only showed significant cytotoxicity at the highest effector: target cell ratios used, at 4 and 7 days after initiation of culture.

*Detection of a cytotoxic supernatant factor or factors from* in vitro *cultured rat LNC*

Investigations into the mechanism of non-specific *in-vitro*-generated cytotoxicity displayed by Mc7-bearer rat

TABLE II.—*Effect of supernatants of cultures of normal and tumour-bearing rat LNC on target-cell growth* in vitro

| Exp. No. | Supernatant[1] dil.: | Target cells[2]—% toxicity: Mc7 | Mc57 | D192A | D23 | 16 day embryos fibroblats |
|---|---|---|---|---|---|---|
| 1 | Fresh N LNC (1/5) | 0 | | −22 | | |
| | Fresh TB LNC (1/5) | −11 | | −16 | | |
| | Cultured N LNC (1/5) | 1 | | −12 | | |
| | Cultured TB LNC (1/5) | 91*** | | 97*** | | |
| 2 | Fresh N LNC (1/5) | 18*** | | | | |
| | Fresh TB LNC (1/5) | 21 | | | | |
| | Cultured N LNC (1/5) | 93*** | | | | |
| | Cultured TB LNC (1/5) | 94*** | | | | |
| 3 | Cultured N LNC (1/5) | 15*** | | | | |
| | Cultured TB LNC (1/5) | 98*** | | | | |
| | Cultured N LNC (1/10) | 16 | | | | |
| | Cultured TB LNC (1/10) | 94*** | | | | |
| | Cultured N LNC (1/20) | 20 | | | | |
| | Cultured TB LNC (1/20) | 82*** | | | | |
| | Cultured N LNC (1/60) | 34 | | | | |
| | Cultured TB LNC (1/60) | 43** | | | | |
| 4 | Cultured N LNC (1/10) | | | | | −23 |
| | Cultured TB LNC (1/10) | | | | | 60*** |
| 5 | Cultured N LNC[3] (1/10) | | | −12 | 22* | |
| | Cultured TB LNC[3] (1/10) | | | 70*** | 99*** | |
| 6 | Cultured N LNC (1/3) | | 32 | −30 | | |
| | Cultured TB LNC (1/3) | | 97*** | 100*** | | |
| 7 | Cultured N LNC (1/12) | −16 | | | | |
| | Cultured TB LNC (1/12) | 91*** | | | | |
| | Cultured N LNC (1/12) 56°C:3 h | 15 | | | | |
| | Cultured TB LNC (1/12) 56°C:3 h | 91*** | | | | |
| 8 | Cultured N LNC (1/3), 2 h SN | | | −11 | | |
| | Cultured N LNC (1/3), 2 h SN | | | 34* | | |

[1] 48 h supernatant from freshly prepared or *in vitro* cultured normal and tumour-bearer rat LNC, unless otherwise stated ((*viz.* 2 h).
[2] Target cells plated at 500 or 1000 cells per well.
[3] Supernatant prepared from cultured rat LNC stored in vapour-phase $N_2$ bank.
*** $P<0{\cdot}001$; ** $P<0{\cdot}01$; * $P<0{\cdot}05$; by Student's $t$ test.

LNC indicated that the reactivity was mediated by a cytotoxic factor or factors released into the culture medium (Table II). Thus supernatants (SN) obtained 48 h after re-culture of cultured TB LNC in fresh medium were highly toxic to Mc7, Mc57, D23, and D192A tumour cells, and to normal embryo fibroblasts. In control experiments, similar SN obtained from fresh normal or TB LNC had little or no cytotoxic activity at the dilutions tested (Table II, Exp. 1 and 2) and except in one experiment (Table II, Exp. 2) SN from cultured normal LNC had minimal activity; a comparative titration of SN from cultured normal and TB LNC is shown in Table II, Exp. 3.

The cytotoxic factor(s) in cultured TB LNC supernatant was shown to be heat-stable (Table II, Exp. 7) and was released from cultured TB LNC within 2 h of re-culture in fresh medium (Table II, Exp. 8). This finding, together with the retention of cytotoxic activity after dilution of supernatant to 1:60 with fresh medium (Table II, Exp. 3), ruled out any possibility of the cytotoxicity being caused by nutrient depletion.

### DISCUSSION

In the present study, LNC from rats bearing a chemically-induced transplantable sarcoma (Mc7) and cultured *in vitro* for 5–7 days without deliberate addition of tumour antigen were shown to be highly cytotoxic for Mc7 target cells. Reactivity was demonstrated using the microcytotoxicity test and assessed by radio-labelling the remaining target cells after a 48h incubation with effector cells. Cytotoxicity could also be seen, and was shown to be cross-reactive with cells derived from a chemically induced rat hepatoma (D202) as well as normal rat fibroblasts. However, cytotoxic events were not detected in either a 3h or 15h $^{51}$Cr-release test. This may reflect an increased susceptibility of adherent target cells to cytotoxic mechanisms, but a more likely explanation is that target cells do not die during the early stages of co-culture of target cells and effector cells. Evidence was obtained that the mechanism of cytotoxicity involved the release of a cytotoxic factor from the cultured TB LNC, and because the release of this factor was rapid, beginning within 2 h of re-culture, the requirement for a long-term cytotoxicity assay would appear to be due to the slow death of target cells after interaction with this factor.

A number of other workers have demonstrated augmented cytotoxicity in cultured lymphocytes. In one series of reports, increased cytolytic activity was found after short-term culture (3–24 h) of lymphocytes from tumour-bearer or tumour-immune animals (de Landazuri & Herberman, 1972; Laux & Lausch, 1974; Vasudevan *et al.*, 1974; Gorczynski & Tigelaar, 1975; Shellam *et al.*, 1976), such *in vitro* activated cells being apparently capable of causing specific tumour rejection *in vivo* (Blasecki & Trevethia, 1975). In the mouse studies, the cytotoxic cells were shown to be sensitive to anti-$\theta$ serum (Vasudevan *et al.*, 1974; Gorczinski & Tigelaar, 1975) and were therefore not NK cells (Herberman & Holden, 1978). This type of activation of cytotoxicity was clearly demonstrable within 24 h of culture, and the cytotoxic cells showed elements of specificity *in vitro* in both short-term (Ortiz de Landazuri & Herberman, 1972; Laux & Lausch, 1974; Vasudevan *et al.*, 1974) and long-term (Wright *et al.*, 1973) cytotoxicity assays.

Burton *et al.* (1977) and Shustik *et al.* (1976) have described a different cytotoxic activity which developed in normal mouse spleen cells over a 5-day culture period and was of broad specificity. Ortaldo *et al.* (1977) made similar observations with human peripheral blood cells, and showed that in this system the cytotoxicity was generated by substances in foetal calf serum, suggesting some kind of mitogenic or antigenic activation. Indeed, these workers, and subsequently others (Callewaert *et al.*, 1978) demonstrated that stimulation of human peripheral blood lymphocytes with phytohaemagglutinin, PPD, or allogeneic cells induced high levels of spontaneous cytotoxicity, which was mediated by an $Fc^+$ cell (Ortaldo *et al.*, 1977). A third type of cytotoxicity developing in culture has been described by Muchmore *et al.* (1977*a*), human peripheral blood cells developing spontaneous cytotoxicity towards red blood cells when cultured alone for 7 days. It was claimed that activation occurred in human serum as well as in foetal calf serum and that the effector cell was $Fc^-$. In our own system, preliminary experiments have shown that foetal calf serum does play a role, in so far as considerable differences in cytotoxicity were found after culture of TB LNC in medium containing different batches of serum (Robins, unpublished observation), but whether this was due to nutritional or antigenic/mitogenic differences between batches of serum is not yet known. It is already clear, however, that there are a number of interesting differences between the phenomenon we have observed and those noted above.

Firstly, we could not detect any cytotoxic activity in a short-term chromium-release assay, whereas in all the other reports reactivity was readily demonstrable in such an assay. This may either reflect a difference in the rate at which different target cells are killed, or indicate that different lytic mechanisms are operative. Secondly, we were able to show that the effector cells in our system released a cytotoxic substance, which was not the case in the systems studied by Burton *et al.* (1977) and Muchmore *et al.* (1977*a*), in which addition of moderate numbers of cold target cells inhibited cytotoxicity. Thirdly, we found that augmentation of cytotoxicity during culture generally occurred only with LNC from tumour-bearer animals and not with LNC from normal animals. Tumour growth therefore played an important part in the induction of the cytotoxic cells. It is interesting to note that the activity of conventional NK cells (Herberman & Holden, 1978) can be augmented by certain *in vivo* treatments, including the inoculation of tumour cells (Herberman *et al.*, 1977; Oehler *et al.*, 1978). However, tumour growth itself has been reported to depress NK-cell activity (Becker & Klein, 1976; Pross & Bains, 1976). Our own studies, using a long-term cytotoxicity assay and target cells derived from solid tumours, have indicated the existence of a second "natural killer" cell, which is characterized by its relatively high affinity for nylon wool (Brooks *et al.*, 1976). The activity of these cells, which is often masked by the growth-promoting activities of other cell types, and is only detectable at high lymphocyte: tumour cell ratios, may be partly mediated by a rapidly released toxic factor, which we have recently shown to be heat-stable (Rees & Brooks, unpublished observation). It is possible that the effector cell in cultured TB LNC is of the same type, being stimulated *in vivo* by the growth of a tumour and revealed by *in vitro* culture either as a result of further stimulation or the loss of some type of suppressor cell. A precedent for this latter postulate has recently been described by Muchmore *et al.* (1977*b*).

The characteristics of the cytotoxic factor we have described are at present unknown, apart from its heat-stability and some preliminary data indicating a mol. wt $>10,000$ (Robins, unpublished). It is therefore not possible to make a meaningful comparison with the legion of cytotoxic factors reported previously in the literature. However, it is worth noting that a malignant cell isolated from human peripheral blood, which had characteristics similar to those of NK cells, released a cytotoxic factor into the culture medium which was claimed to have anti-tumour activity *in vivo* (Karpas, 1978). Whether the factor described in the present study is also active *in vivo* remains to be determined.

The authors wish to thank Miss J. McVeagh, Mr O. F. H. Roberts and Mrs M. E. Addison for their skilful technical assistance.

This work was supported by a grant from the Cancer Research Campaign.

REFERENCES

BALDWIN, R. W. & BARKER, C. (1967) Demonstration of tumour-specific humoral antibody against aminoazo dye-induced rat hepatoma. *Br. J. Cancer*, **21**, 793.

BECKER, S. & KLEIN, E. (1976) Decreased "natural killer" effect in tumour-bearing mice and its relation to the immunity against oncornavirus-determined cell surface antigens. *Eur. J. Immunol.*, **6**, 892.

BLASECKI, J. W. & TREVETHIA, S. S. (1975) Restoration of specific immunity against SV40 tumour-specific transplantation antigen to lymphoid cells from tumour-bearing mice. *Int. J. Cancer*, **16**, 275.

BROOKS, C. G., REES, R. C. & BALDWIN, R. W. (1976) Studies on the microcytotoxicity test. I. Evidence that the effects of normal lymphoid cells on tumour cell growth in micro-test plates may be caused by non-immunological modifications of the culture medium. *Int. J. Cancer*, **18**, 778.

BROOKS, C. G., REES, R. C. & ROBINS, R. A. (1978) Studies on the microcytotoxicity test. II. The uptake of amino acids ([$^{3}$H] leucine or [$^{75}$Sc] methionine) but not nucleosides ([$^{3}$H] thymidine or [$^{125}$I] IUdR) or $^{51}GO_4^{2-}$ provides a direct and quantitative measure of target cell survival in the presence of lymphoid cells. *J. Immunol. Meths.*, **21**, 111.

BURTON, R. C., CHISM, S. E. & WARNER, N. L. (1977) *In vitro* induction of tumour specific immunity. VII. Does autosensitization occur with *in vitro* culture of T lymphocytes? *J. Immunol.*, **119**, 1329.

CALLEWAERT, D. M., LIGHTBODY, J. J., KAPLAN, J., JAROSZEWSKI, J., PETERSON, W. D. & ROSENBERG, J. C. (1978) Cytotoxicity of human peripheral lymphocytes in cell-mediated lympholysis; antibody-dependent cell-mediated lympholysis and natural cytotoxicity assays after mixed lymphocyte culture. *J. Immunol.*, **121**, 81.

CHISM, S. E., BURTON, R. C. & WARNER, N. L. (1976) *In vitro* induction of tumour-specific immunity. II. Activation of cytotoxic lymphocytes to murine oncofoetal antigens. *J. Natl Cancer Inst.*, **57**, 377.

GORDON, R. D., SIMPSON, E. & SAMELSON, L. E. (1975) *In vitro* cell-mediated immune response to the male specific (H-Y) antigen in mice. *J. Exp. Med.*, **142**, 1108.

GORCZYNSKI, R. M. & TIGELAAR, R. E. (1975) Cell-mediated immunity to murine tumour allografts. Increase in the activities of activated thymus-derived cells following *in vitro* incubation. *Cell Immunol.*, **18**, 121.

HERBERMAN, R. B. & HOLDEN, H. T. (1978) Natural cell-mediated immunity. *Adv. Cancer Res.*, **27**, 305.

HERBERMAN, R. B., NUNN, M. E., HOLDEN, H. T., STAAL, S. & DJEU, J. Y. (1977) Augmentation of natural cytotoxic reactivity of mouse lymphoid cells against syngeneic and allogeneic target cells. *Int. J. Cancer*, **19**, 555.

KALL, M. A. & HELLSTRÖM, I. (1975) Specific stimulatory and cytotoxic effects of lymphocytes sensitized *in vitro* to either alloantigens or tumour antigens. *J. Immunol.*, **114**, 1083.

KARPAS, A. (1978) A humoral cytotoxic substance produced by a human killer cell line. *Br. J. Cancer*, **36**, 437.

KUPERMAN, O., FORTNER, G. W. & LUCAS, Z. J. (1975*a*) Immune response to a syngeneic mammary adenocarcinoma. II. *In vitro* generation of cytotoxic lymphocytes. *J. Immunol.*, **115**, 1277.

KUPERMAN, O., FORTNER, G. W. & LUCAS, Z. J. (1975*b*) Immune response to a syngeneic mammary adenocarcinoma. III. Development of memory and suppressor functions modulating cellular cytotoxicity. *J. Immunol.*, **115**, 1282.

LAUX, D. & LAUSCH, R. N. (1974) Reversal of tumour-mediated suppression of immune reactivity by *in vitro* incubation of spleen cells. *J. Immunol.*, **112**, 1900.

MUCHMORE, A. V., DECKER, J. M. & BLAESE, R. M. (1977*a*) Spontaneous cytotoxicity of human peripheral mononuclear cells towards red blood cell targets *in vitro*. I. Characterization of the killer cell. *J. Immunol.*, **119**, 1680.

MUCHMORE, A. V., DECKER, J. M. & BLAESE, R. M. (1977*b*) Spontaneous cytotoxicity of human peripheral mononuclear cells towards red blood cell targets. II. Time-dependent loss of suppressor cell activity. *J. Immunol.*, **119**, 1686.

OEHLER, J. R., LINDSEY, L. R., NUNN, M. E. & HERBERMAN, R. B. (1978) Natural cell-mediated cytotoxicity in rats. II. *In vivo* augmentation of NK-cell activity. *Int. J. Cancer*, **21**, 210.

ORTALDO, J. R., BONNARD, G. D. & HERBERMAN, R. B. (1977) Cytotoxic reactivity of human lymphocytes cultured *in vitro*. *J. Immunol.*, **119**, 1351.

ORTIZ DE LANDAZURI, M. & HERBERMAN, R. B. (1972) *In vitro* activation of cellular immune response to gross virus-induced lymphoma. *J. Exp. Med.*, **136**, 969.

PROSS, H. F. & BAINES, M. G. (1976) Spontaneous human lymphocyte-mediated cytotoxicity against tumour target cells. I. The effect of malignant disease. *Int. J. Cancer*, **18**, 593.

SHELLAM, G. R., KNIGHT, R. A., MITCHISON, N. A., GORCZYNSKI, R. M. & MAOZ, A. (1976) The specificity of effector T cells activated by tumours induced by murine oncornavirus. *Transplantation*, **29**, 249.

SHUSTIK, C., COHEN, I. R., SCHWARTZ, R. S., LATHAM-GRIFFIN, E. & WAKSAL, S. D. (1976) T lymphocytes with promiscuous cytotoxicity. *Nature*, **263**, 699.

SIMPSON, E. R., GORDON, M., TAYLOR, J. M. & CHANDLER, P. (1975) Micromethods for induction and assay of mouse lymphocyte reactions and cytotoxicity. *Eur. J. Immunol.*, **5**, 451.

VASUDEVAN, D. M., BRUNNER, K. T. & CEROTTINI, J. C. (1974) Detection of cytotoxic T lymphocytes in the EL4 mouse leukaemia system: increased activity of immune spleen and peritoneal cells following pre-incubation and cell fractionation procedures. *Int. J. Cancer*, **14**, 301.

WAGNER, H., HARRIS, A. W. & FELDMANN, M. (1972) Cell-mediated immune response *in vitro*: II. The role of thymus and thymus-derived lymphocytes. *Cell. Immunol.*, **4**, 39.

WRIGHT, P. W., ORTIZ DE LANDAZURI, M. & HERBERMAN, R. B. (1973) Immune response to Gross virus-induced lymphoma: Comparison of two *in vitro* assays of cell-mediated immunity. *J. Natl Cancer Inst.*, **50**, 947.